# Conduit CAR: Redirecting CAR T-Cell Specificity with A Universal and Adaptable Bispecific Antibody Platform

**DOI:** 10.1158/2767-9764.CRC-21-0150

**Published:** 2022-03-22

**Authors:** M. Jack Borrok, Yonghai Li, Paul B. Harvilla, Bharathikumar Vellalore Maruthachalam, Ninkka Tamot, Christine Prokopowitz, Jun Chen, Sathya Venkataramani, Iqbal S. Grewal, Rajkumar Ganesan, Sanjaya Singh

**Affiliations:** 1 Janssen BioTherapeutics, Spring House, Pennsylvania.; 2 M. Jack Borrok and Yonghai Li contributed equally to this article.

## Abstract

The success of chimeric antigen receptor (CAR) T-cell therapy against hematologic malignancies has altered the treatment paradigm for patients with these diseases. Nevertheless, the occurrence of relapse due to antigen escape or heterogeneous antigen expression on tumors remains a challenge for first-generation CAR T-cell therapies as only a single tumor antigen can be targeted. To address this limitation and to add a further level of tunability and control to CAR T-cell therapies, adapter or universal CAR T-cell approaches use a soluble mediator to bridge CAR T cells with tumor cells. Adapter CARs allow simultaneous or sequential targeting of multiple tumor antigens, control of immune synapse geometry, dose control, and the potential for improved safety. Herein, we described a novel CAR T-cell adapter platform that relies on a bispecific antibody (BsAb) targeting both a tumor antigen and the GGGGS (G_4_S) linker commonly used in single-chain Fv (ScFv) domains expressed on CAR T-cell surfaces. We demonstrated that the BsAb can bridge CAR T cells to tumor cells and potentiate CAR T-cell activation, proliferation, and tumor cell cytolysis. The cytolytic activity of CAR T-cells was redirected to different tumor antigens by changing the BsAb in a dose-dependent manner. This study highlights the potential of G_4_S-displaying CAR T cells to be redirected to engage alternative tumor-associated antigens (TAA).

**Significance:**

New approaches are needed to address relapsed/refractory disease and manage potential toxicities associated with CAR T-cell therapy. We describe an adapter CAR approach to redirect CAR T cells to engage novel TAA-expressing cells via a BsAb targeting a linker present on many clinical CAR T-cell therapeutics. We anticipate the use of such adapters could increase CAR T-cell efficacy and reduce potential CAR-associated toxicities.

## Introduction

New methods for redirecting and reengineering T cells have revolutionized the cancer treatment paradigm by harnessing the vast potential of the human immune system (1). Chimeric antigen receptor (CAR) T-cell immunotherapy has shown tremendous success in the treatment of acute lymphocytic leukemia (ALL) and refractory diffuse large B-cell lymphoma (DLBCL) led by the FDA-approved CD19 targeting CARs Kymriah (tisagenlecleucel) and Yescarta (axicabtagene ciloleucel; refs. 2, 3). Building on the clinical success of these pioneering therapies, dozens of novel CAR therapies are currently under clinical and preclinical evaluation for not only hematologic malignancies, but solid tumors as well (4).

Despite their initial success, multiple challenges remain for CAR T-cell therapies (5). Understanding and overcoming multiple different potential relapse mechanisms is a central challenge for CAR T-cell therapies (6, 7). Mechanisms for relapse in current CAR T-cell therapies include antigen escape via various methods, defective T-cell function such as anergy, activation induced cell death, and difficulty overcoming suppressive tumor microenvironments (8–10). In addition to various resistance mechanisms, targeting solid tumors via CAR T-cell therapy poses the additional challenges of increased antigen heterogeneity, immunosuppressive tumor environments, and accessibility (4, 11). Furthermore, even efficacious CAR T-cell therapies can be associated with serious adverse events including cytokine release syndrome (CRS), and immune effector cell–associated neurotoxicity syndrome (ICANS) among others (12).

Numerous approaches are under investigation to increase CAR T-cell efficacy in solid tumor settings, reduce CAR T-cell relapse rates, and reduce the toxicity associated with CAR T-cell therapy. Many of these approaches involve changing the prevailing mono-specific design of CAR constructs to make them more modular and adaptable. Although CAR design can vary, the majority of CARs in the clinic include an extracellular domain which recognizes a tumor-associated antigen (TAA). The extracellular binding domain is usually composed of a single-chain Fv (ScFv) region derived from an antibody. ScFv molecules are composed of a variable heavy (VH) and a variable light (VL) domain derived from an antibody connected by an unstructured synthetic linker [typically made of repeating GGGGS (G_4_S) repeats]. Attached to the extracellularly accessible CAR ScFv are the hinge region and transmembrane domain that are commonly derived from the CD8 extracellular domain. Intracellularly, the CAR protein contains one more costimulatory domains and the intracellular signaling domain from CD3ζ. A drawback of this mono-specific CAR T-cell design is that antigen escape can render CAR T cells unable to recognize the tumor and may lead to relapse.

The CAR–adapter approach (13) is designed to allow for controllable tumor engagement to reduce toxicity and increase adaptability to engage multiple antigens (14). This approach involves decoupling antigen recognition the CAR T-cell itself and using a soluble mediator to act as a bridge or “conduit” between the engineered T-cell and the targeted tumor cell. This design relies on recognition of the soluble adapter by the extracellular portion of the CAR. A central feature of the CAR–adapter approach is the introduction of an engineered antigen on T cells that can be engaged by the soluble adapter molecule (to bridge the T cell with tumor cells). Multiple designs for CAR–adapter pairs have been put forward with a variety of engineered antigens and respective adapters including an antifluorescein CAR with fluorescein-labeled antibodies (15–17), CD16-based CARs designed to bind to antibody Fc regions (18–20), CAR–adapter pairs based on leucine zippers (21), and a CAR–adapter pair using the peptide neo-epitope (PNE) derived from the yeast transcription factor GCN4 and an antibody recognizing it (17, 22).

**FIGURE 1:**
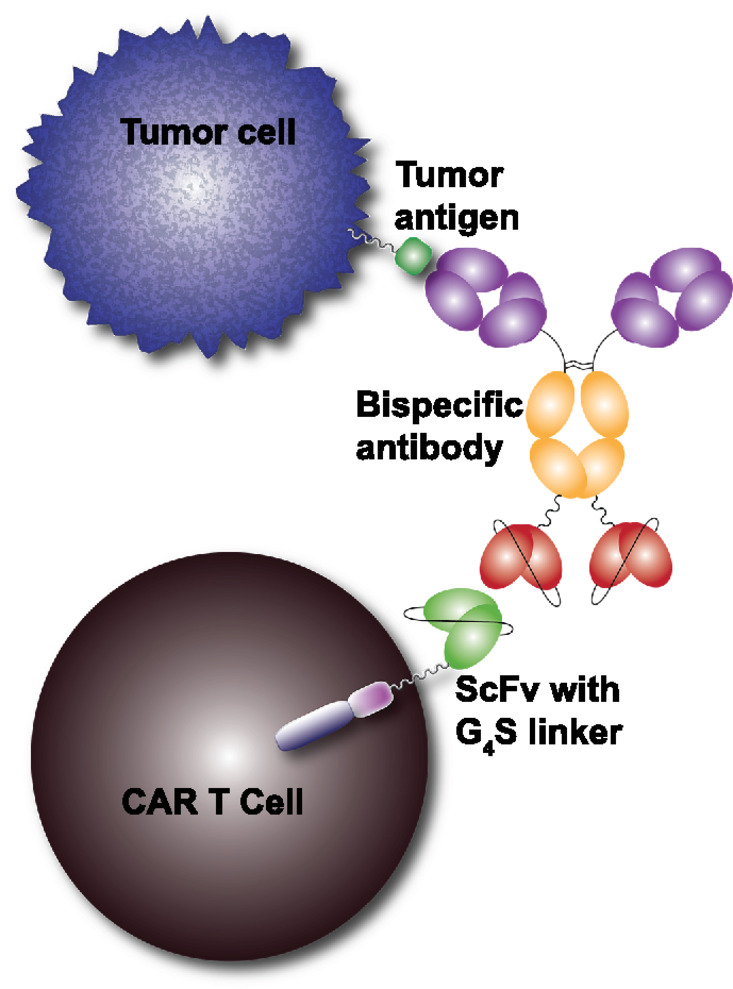
Schematic illustration of the universal CAR T-cell platform. The extracellular portion of the designed CAR construct contains an exposed (G_4_S)_4_ linker sequence joining the VH and VL regions of the TAA targeting ScFv. The (G_4_S)_4_-containing CAR molecules can act as a functional epitope and be recognized by anti-G_4_S binding antibody. Using a bispecific antibody, tumor cells expressing a TAA can be targeted via the adapter bispecific. This bispecific adapter molecule then redirects CAR T cells to a new tumor cell target and consequently activate CAR T cells to further engage tumor cells.

In this study, we describe a versatile CAR–adapter pair that does not rely on the introduction of any novel engineered antigen on T cells, but rather exploits an existing feature already present in most clinical CAR T-cell therapies, the flexible ScFv linker. (Fig. 1). We generated an antibody targeting the (GGGGS)_n_ or (G_4_S)_n_ linker present on most existing CARs and have used BsAbs targeting both this linker and a TAA to demonstrate killing of cells expressing the TAA. We showed that CAR T cells expressing either a germline antibody ScFv (with no known specificity) or a CD19 targeting CAR can be redirected to target prostate tumor cells via a bispecific adapter molecule. We anticipate this approach may be clinically advantageous due to its adaptability to target novel TAAs, control of potential toxicity via dosing adjustment, and its potential compatibility with existing clinical CARs.

## Materials and Methods

### Reagents and Cell Lines

Human prostate tumor cell lines were purchased from ATCC; these include PC3 (RRID:CVCL_0035; obtained and banked in 2019) and LNCaP (RRID:CVCL_0395; obtained and banked in 2018) cells. The PC3 cells used in this article were sorted and enriched for prostate-specific membrane antigen (PSMA) surface expression prior to use. HEK293T (ATCC; obtained and banked in 2018) cells were maintained in DMEM supplemented with 10% heat-inactivated FBS in purpose to generate lentiviral particles. Cell lines used in this study were banked immediately after receiving and experiments occurred within approximately 2 months of resuscitation; cell lines were not authenticated. All cells were periodically tested for mycoplasma at the using MycoAlert (Lonza) and found to be negative. G_4_S peptides of various lengths were generated by peptide synthesis techniques at New England Peptide company. A biotin moiety was appended to the C-terminal end of the peptides to allow for immobilization on streptavidin coated ELISA plates or beads.

The following mAbs used in the study were all purchased from BD Biosciences: APC-Cy7 mouse anti-human CD3; FITC-anti human CD8; PE-Cy7 anti-human CD4; PE-anti CD107a; PE-anti human Fc mAb; myc AF-647. Protein A–coated magnetic beads were purchased from Spherotech, and recombinant human CD19-Fc fusion (rhCD19) was purchased from BioLegend. Protein A–coated beads were loaded with Fc-containing bispecifics or human CD19-Fc according to manufacturer's instructions and excess soluble protein was washed before use.

## Antibody Generation

Antibodies were generated by immunizing New Zealand white rabbits. Rabbits were manually restrained and the dorsal area shaved and cleaned with chlorhexidine. Immunogen (G_4_S peptide-BSA) was injected in 4–8 sites subcutaneously (usually 0.25 mL/site) or intradermally. The animals were immunized 1–2 times based on serum titer. Subsequently, the animals were anesthetized, blood collected, and the spleen and secondary lymph nodes aseptically removed for fusion for hybridoma generation or subjected to single B-cell sorting via FACS. Hybridoma supernatants were screened by MSD electrochemiluminescence for binding to biotinylated peptide. Hits with the desired binding profile were selected and sequenced as described below.

## Variable Region Cloning

Both RNA purified by Qiagen Kit (RNeasy Plus Mini Kit) and B cells lysate were used for cDNA synthesis using the Smarter cDNA synthesis kit (ClonTech). To facilitate cDNA synthesis, oligo(dT) was used to prime reverse transcription of all messenger RNAs followed by “5′ capping” with a Smarter IIA oligonucleotide. Subsequent amplification of the VH and VL fragments was performed using a 2-step PCR amplification using 5′ primers targeting the Smarter IIA cap and 3′ primers targeting consensus regions in CH1. Briefly, each 50 μL PCR reaction consisted of 20 μmol/L of forward and reverse primer mixes, 25 μL of PrimeStar Max DNA polymerase premix (ClonTech), 2 μL of unpurified cDNA, and 21 μL of double distilled H2O. The cycling program started at 94°C for 3 minutes, followed by 35 cycles (94°C for 30 seconds, 55°C for 1 minute, 68°C for 1 minutes), and a final extension reaction at 72°C for 7 minutes. The second round PCR was performed with VL and VH second round primers containing 15-bp complementary extensions that “overlap” respective regions in their respective Lonza mother vector (VH and VL). Second round PCR was performed with the following program: 94°C for 3 minutes; 35 cycles (94°C for 30 seconds, 55°C for 1 minutes, 68°C for 1 minutes), and a final extension reaction at 72°C for 7 minutes. In-Fusion HD Cloning Kit (ClonTech) was used for directional cloning of VL gene into Lonza huIgKappa or Lambda vector and VH gene into Lonza huIgG1 vector. To facilitate In-Fusion HD cloning, PCR products were treated with Cloning Enhancer before In-Fusion HD Cloning. Cloning and transformation were performed according to manufacturer's protocol (ClonTech). Mini-prep DNAs were subjected to Sanger sequencing to confirm that complete V-gene fragments were obtained.

G_4_S binding antibodies identified from hybridoma supernatants were screened for binding to G_4_S containing peptides as well as ScFvs containing G_4_S linkers between VH and VL domains. To demonstrate specificity, antibodies were counterscreened against another flexible glycine-rich linker, the modified Bird and colleagues’ linker (23).

## ELISA Methods

ScFvs with (G_4_S)_4_ and modified Bird and colleagues linker (23) were coated on 96-well maxisorp plates (Nunc) overnight at 4°C (2 μg/mL in PBS). Plates were then blocked with 5% milk in PBS for 1 hour at room temperature. After washing (3×), titrated antibodies were added and allowed to incubate for 1.5 hour. After three washes in PBS, peroxidase-conjugated goat anti-human Fc (Jackson Immunoresearch) was used for detection (1-hour incubation). The wells were washed three times, and TMB substrate solution was added. The reaction was stopped with 2 N H_2_SO_4_ and the absorbance at 450 nm was measured. Data analysis was performed using GraphPad Prism 7.0 (GraphPad Prism, RRID:SCR_002798).

## Flow Cytometry

HEK-293 cells expressing CARs with either (G_4_S)_3_ or the modified Bird and colleagues’ linker (23) were harvested and the pellet suspended in DPBS. Cells were plated at 100,000 cells/well in a round-bottom 96-well plate (Nunc) and washed 3× with DPBS. Live/Dead stain (Live/Dead near IR, Thermo Fisher) was then added following manufacturer's directions and cells were washed again with PBS. Titrated primary antibody was then added in FACS Buffer (BD Biosciences) to wells and cells incubated at 4°C for 30 minutes in the dark. Cells were washed 2× with FACS Buffer and secondary antibody [anti-human Fc PE; (LifeSpan) catalog no. LS-C68730–500, RRID:AB_1653937] was added (1:500). Cells were again incubated at 4°C for 30 minutes in the dark. After washing 3× with FACS buffer, Flow cytometry was performed using Intellicyt IQue2 (Sartorius). Analysis was performed using Intellicyt software and data were graphed and analyzed using GraphPad Prism 7.0.

## Biolayer Inferometry

Biolayer Inferometry was performed using the Octet Red system (ForteBio). Streptavadin Dip and Read biosensors were prewetted by dipping them into Octet Kinetic Buffer (ForteBio) for 10 minutes before use and they were incubated with various Biotin–G_4_S peptides and immobilized on sensor tips. Tips were then dipped into wells with varying concentrations of anti-G_4_S antibodies after 170-second loading time, tips were dipped into Octet Kinetic Buffer for a disassociation step (170 seconds). Sensograms were recorded and analyzed using Octet Software to determine on-rate and off-rate.

### BsAb Cloning, Expression, and Purification

Bispecific mAbs targeting two different prostate cancer antigens were generated in the study [anti- (G_4_S)_n_ × anti-PSMA and anti-(G_4_S)_n_ × anti-TMEFF2] using the bispecific format described in Coloma and colleagues (24). ScFv portions of the bispecific antibodies were generated using 20 amino acid–long linkers modified from Bird and colleagues (23) with the following amino acid sequence: GGSEGKSSGSGSESKSTGGS. G_4_S linkers were not used in the construction of these bispecifics to avoid intramolecular binding and self-aggregation. DNA gBlocks were synthesized containing the sequence of anti- (G_4_S)_n_ scFv or anti-PSMA scFv or anti-TMEFF2 scFv. These gBlocks were inserted into a mammalian expression vector using InFusion method. Human CD4 signal peptides were encoded to allow for efficient secretion of antibodies into culture supernatant. All constructs were sequence verified and scaled up using Endotoxin free maxi preparation kits. ExpiCHO mammalian expression system was used for protein expression (Invitrogen). To ensure proper light chain loading in the mature protein, a 3:1 light chain:heavy chain DNA ratio was used. The DNA mixture was incubated with Expifectamine and immediately added to the culture. ExpiCHO suspension cultures were harvested after 10 days by centrifuging at 3,000 × *g* for 10 minutes to pellet cells. The supernatants were stored at 4°C until purification.

HiTrap MabSelect Sure (GE Healthcare) columns were used to purify BsAb protein. Supernatants from transfected cells were applied to the column at a flow rate of 1 mL/minute for maximum capture. Columns were washed using 20 column volumes of PBS until a clean baseline was obtained as monitored by UV A_280_. Fractions were eluted with 100 mmol/L glycine-HCl pH 2.8 and neutralized with 100 mmol/L Tris-HCl pH 8.0. Eluted fractions were then tested for the presence of recombinant bispecific protein using nondenaturing and denaturing SDS-PAGE gels (Bio-Rad) and samples with similar properties were pooled, dialyzed in PBS, and again assessed by SDS-PAGE analysis (Supplementary Fig. S1) and were stored at 4°C until use.

## CAR Construction, Lentiviral Production, and CAR T-Cell Generation

Human codon–optimized DNA comprising the 3–23/B3 ScFv sequence, CD8α hinge, and transmembrane domains, 4–1BB, and CD3ξ domain were cloned into the lentiviral vector. An additional CD19 targeting CAR with an N-terminal MYC-tag and a (G_4_S)_3_ ScFv linker was also constructed as above. To produce high-titer replication-defective lentiviral vectors, 293-T human embryonic kidney cells were transfected with lentiviral packaging plasmids [pVSV-G (RRID:Addgene_138479), pRSV.REV (RRID:Addgene_106453), pMDLg] and CAR-encoding lentiviral vector using Lipofectamine 2000 (Invitrogen). The viral supernatant was harvested at 24 and 48 hours posttransfection. Viral particles were concentrated using Lenti-X concentrator (Takara). Concentrated viral particles were resuspended in PBS and stored frozen at −80°C. Primary human CD4^+^ and CD8^+^ T cells (purchased from HemaCare) were isolated from healthy volunteer donors following leukapheresis by negative selection. T cells were cultured in complete media (RPMI1640 supplemented with 10% heat-inactivated FBS, 100 U/mL penicillin, 10 mmol/L HEPES), stimulated with anti-CD3 and anti-CD28 mAb-coated beads (Invitrogen). Twenty-four hours after activation, T cells were transduced with recombinant lentiviral particles at multiplicity of infection of approximately 5–10. Human recombinant IL2 (PeproTech) was added every other day to 50 IU/mL final concentration and 0.5–1 × 10^6^ cells/mL cell density was maintained. CAR surface expression was verified by flow cytometry using GLPB30 IgG as primary staining followed by PE-labeled anti-human Fc antibody as secondary antibody. Anti-MYC antibody labeled with AF-647 (BioLegend) was used to stain anti-CD19 MYC–tagged CAR T cells and assess expression levels.

### CAR T-Cell CD107a Assay and Proliferation Assay

CD107a (also known as LAMP-1) is a marker of CD8^+^ T-cell degranulation following stimulation and activation (25). For CD107a assay, CAR T cells were cocultured with PC3 prostate tumor cells in a 96-well plate at an effector-to-target ratio (E:T) equal to 5:1 in the presence or absence of anti-PSMA × anti-G_4_S BsAbs (5 mg/mL). Phycoerythrin-labeled anti-CD107a antibody was added 1 hour before adding protein transport inhibitor, Golgi Stop (BD Biosciences) and the plate was incubated for 3 hours. The anti-CD8 antibody was added and incubated at 37°C for 30 minutes. After incubation, the samples were washed once and subjected to flow cytometry. The data were analyzed by FlowJo (FlowJo, RRID:SCR_008520) software. For the T-cell proliferation assay, CAR T cells were prelabeled with 5 mmol/L CFSE (Invitrogen) according to the manufacturer's protocol. CAR T cells were cocultured with PC3 prostate tumor cells at an E:T ratio of 1:1 in a 96-well round-bottom plate in 200 μL RPMI complete media. The BsAbs of anti-PSMA × anti-G_4_S (5 μg/mL) were added. After a 3-day incubation, T cells were stained with anti-CD3 mAb and analyzed for CFSE distribution.

### Cytotoxicity Assays by xCELLigence

Cytotoxicity was measured in a real-time cell analyzer xCELLigence (Roche) using adherent tumor cell lines as target cells. All experiments were performed using the respective target cell culturing media. Fifty microliters of medium was added to E-Plates 96 (Roche) for measurement of background values. Target cells used in the experiments include PC3 cells (enriched for PSMA expression) and LNCaP tumor cell lines. Target cells were seeded in an additional 100 μL medium at a density of 10,000 cells per well. Suitable cell densities were determined by previous titration experiments. Cell attachment was monitored using the RTCA SP (Roche) instrument and the RTCA software Version 1.1 (Roche) until the plateau phase was reached.

At approximately 19 hours after seeding with TAA bearing target cells, CAR T cells were added at different E:T ratios ranging from 20:1 to 1:1. Various concentrations of BsAb were also added at this time at concentrations ranging from 0.2 to 20 μg/mL. Upon addition of effector cells, impedance measurements were performed every 15 minutes for up to 81 hours. All experiments were performed in triplicates. Changes in electrical impedance were expressed as a dimensionless cell index (CI) value, which derives from relative impedance changes corresponding to cellular coverage of the electrode sensors, normalized to baseline impedance values with medium only. To analyze the acquired data, CI values were exported, and percentage of lysis was calculated in relation to the control cells lacking any CAR T cells and 1% Triton X-100–treated cells (100% lysis). The percentage of cytolysis was readily calculated using this formula: percentage of cytolysis = [(normalized cell index with no CAR T cell/no treatment – normalized cell index with CAR T cell/and treatment)/normalized cell index with no CAR T cell/no treatment] × 100.

Cytotoxicity of the CAR-expressing T cells was also tested by using the IncuCyte zoom living cell imaging system. Coculture was set up the same way as above in xCELLigence. Assay images were taken every 30 minutes and the number of dead cells was quantified.

### Cytokine Assay (Intellicyt iQue)

The Intellicyt human T-cell activation and cytokine profiling kit was applied for T-cell activation and cytokine profile. Briefly, CAR T cells were cocultured with PC3 prostate tumor cells at an E:T ratio of 1:1 in 96-well round-bottom plate in 200 μL RPMI complete media. The BsAbs of anti-PSMA × anti-G4S (5 mg/mL) was added. Coculture without BsAb as well as target and CAR T cells alone were used as controls. Seventy-two hours later, T-cell activation was assessed by the TCA kit from a 30 μL cell/supernatant mixture sample following the protocol. Samples were acquired on the Intellicyt iQue Screener PLUS. Standard curves were generated to quantitate the levels of secreted cytokines. Data were analyzed with ForeCyt software. In another experiment, BsAb- or rhCD19-coated beads were substituted for PC3 tumor cells and used to activate CAR T cells (5:1 bead:cell ratio was used).

### Data Availability Statement

Data were generated by the authors and are included in the article or available upon request.

## Results

### Generation of an Anti-(G4S)_n_ Linker Antibody

**FIGURE 2:**
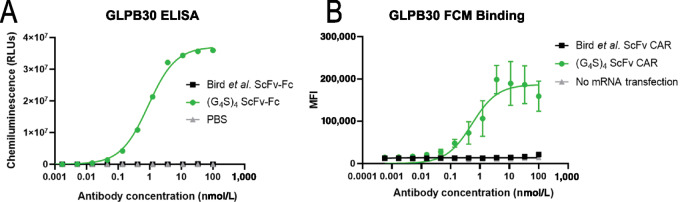
Specificity of the GLPB30 antibody for (G_4_S)_n_ linkers. **A,** ELISA showing binding of GLPB30 anti-G_4_S antibody to immobilized scFv containing a (G_4_S)_4_ linker (green circles) and no binding to an immobilized non-G_4_S linker containing ScFv with the modified Bird and colleagues’ linker (ref. 21; black squares) or uncoated wells (gray triangles). GLPB30 demonstrated dose-dependent binding and an EC_50_ of 0.57 nmol/L. **B,** GLPB30 binding to HEK293T cells expressing an ScFv with an (G4S)_4_ linker observed by flow cytometry (green circles). No binding is observed with HEK293T cells expressing an ScFv with a non-G4S linker (black squares) or to untransfected cells (gray triangles). GLPB30-bound cell surface antigen in a dose -dependent manner with a calculated EC_50_ of 0.78 nmol/L.

To develop a modular T-cell adapter that can bind to a variety of TAA-binding domains, we sought to generate antibodies that recognize the poly-Glycine-Serine linker peptide because of its ubiquitous use in ScFv constructs. Hybridoma screening of clones against (G_4_S)_4_ containing ScFvs, yielded an antibody (GLPB30) with high affinity to the (G_4_S)_4_ linker. To assess the specificity of GLPB30, ELISA binding assays were performed using ScFvs containing (G_4_S)_4_ linker and ScFvs containing an alternate 20 amino acid linker–modified form of the Bird and colleagues (23) ScFv linker (GGSEGKSSGSGSESKSTGGS). GLPB30 bound to immobilized ScFv containing the (G_4_S)_4_ linker with an EC_50_ of 0.57 nmol/L but did not bind to a ScFv (with the same variable domains) containing the modified Bird and colleagues’ linker (Fig. 2A). Similar results were observed when these ScFvs were generated as CAR constructs and binding to GLPB30 was assessed by flow cytometry (Fig. 2B). GLPB30 bound to HEK 293 cells expressing the (G_4_S)_4_ linker containing ScFv with an EC_50_ of 0.78 nmol/L. No binding was observed to HEK 293 cells expressing CAR ScFvs with a different linker (Fig. 2B).

The minimal length of (G_4_S)_n_ peptide that GLPB30 could recognize was then determined. G_4_S peptides of differing lengths (from 5 to 20 AA in length) were generated and binding was assessed via biolayer interferometry (BLI; Table 1). No binding was observed to (G_4_S) peptides below 10 AA in length [i.e.*,* shorter than (G_4_S)_2_]. Binding could be observed to the 10 amino acid–long linker (G_4_S)_2_ albeit with lower affinity than to the (G_4_S)_4_ peptide. At approximately 16 AA in length, binding was similar to that of the 20 amino acid (G_4_S)_4_ peptide and leveled off. BsAbs generated with GLPB30 variable domains exhibited a similar binding pattern to that of GLPB30 IgG.

**Table 1:** Binding of GLPB30 variants to various length G_4_S peptides determined by BLI.

Peptide (AA length)	mAb *K*_D_ (nmol/L)	BsAb1 *K*_D_ (nmol/L)	BsAb2 *K*_D_ (nmol/L)
GGGGSGGGGGSGGGGSGGGGS (20)	3.6	0.4	1.6
GGGGSGGGGGSGGGGSGGGG (19)	2.4	0.4	1.6
GGGGSGGGGGSGGGGSGGG (18)	2.5	0.2	1.3
GGGGSGGGGGSGGGGSGG (17)	2.5	0.2	1.3
GGGGSGGGGGSGGGGSG (16)	4.0	0.5	3.9
GGGGSGGGGGSGGGGS (15)	8.2	3.1	13.7
GGGGSGGGGGSGGGG (14)	9.4	6.4	21.4
GGGGSGGGGGSGGG (13)	7.9	5.7	25.9
GGGGSGGGGGSGG (12)	8.1	5.7	27.4
GGGGSGGGGGSG (11)	15.6	7.3	41.7
GGGGSGGGGGS (10)	18.3	7.4	40.0

NOTE: Binding was determined for GLPB30 formatted as a human IgG1 mAb, a BsAb with GLPB30 as the ScFv fused to the IgG1 C-terminus (BsAb2) or as the Fab arms of a BsAb containing a C-terminal ScFv (BsAb1). No binding was observed for G_4_S peptides shorter than 10 amino acids in length.

### Generation of a Germline ScFv CAR Construct

**FIGURE 3:**
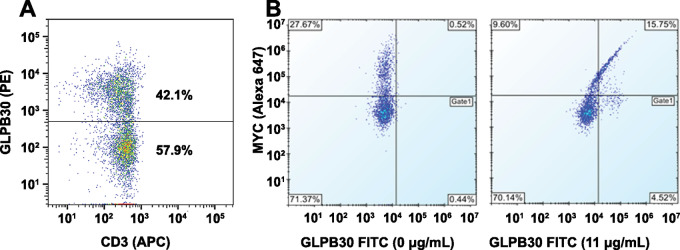
Cell binding of GLPB30 antibodies to CAR T-cell constructs containing G_4_S-linked ScFvs observed by flow cytometry. **A,** 3–23/B3 CAR-transgene expression was detected by anti-G_4_S GLPB30 antibody followed by anti-human PE secondary antibody. Staining was performed two days following mRNA transduction of CD3^+^ pan T cells. **B,** Anti-CD19 CAR with an (G_4_S)_3_ linker containing an N-terminal MYC tag could be detected on lentiviral-transfected pan T cells. MYC-positive CAR T cells had increased GLPB30 staining whereas MYC-negative cells had little GLPB30 staining.

We hypothesized that a BsAb targeting both the (G4S)_n_ linker on T cells expressing CARs and a TAA could facilitate a cytotoxic T-cell response similar to traditional CAR T cells that directly engage TAAs. To assess this hypothesis, we generated a lentiviral CAR construct encoding the human germline antibody 3–23/B3 as an ScFv containing the (G_4_S)_4_ linker (26, 27). The human germline 3–23/B3 ScFv (with the human 3–23 gene encoding the heavy chain and B3 for the light chain) is not expected to have specificity to TAAs as these antibodies have not undergone somatic mutations in response to specific antigen presentation. The CAR construct consists of 3–23/B3 ScFv linked to a human CD8α hinge and transmembrane region, followed by the combination of CD3ζ and 4–1BB signaling moiety forming a second-generation CAR. Both CAR-expressing lentiviral-transduced pan-T cells as well as pan-T cells transfected via CAR-encoding mRNA were generated. The extracellular (G_4_S)_4_ ScFv linker could be detected via flow cytometry analysis utilizing the human GLPB30 antibody and a PE-labeled anti-human secondary antibody (Fig. 3A). In addition to binding the (G_4_S)_4_ containing 3–23/B3 3–23/B3CAR, GLPB30 binding was also demonstrated to pan-T cells that had been transduced via lentivirus expressing an anti-CD19 CAR with a (G_4_S)_3_ linker and a N-terminal MYC tag. GLPB30 costained with MYC-positive CAR T cells (Fig. 3B).

## Design and Characterization of Bispecific Conduit Molecule

BsAbs were then generated to test whether targeting the G_4_S linker in ScFvs could act as effective adapter molecule and facilitate CAR T-cell signaling and cytotoxicity. ScFv fusions linked directly to the C-terminus of IgG heavy chains (24) were employed to generate the bispecific adapters. These molecules were generated both with the anti-TAA binding moiety as the antibody Fab arm and the GLPB30 antibody as the ScFv arm and in the reverse orientation (with the GLPB30 variable domains formatted as a Fab arm and the anti-TAA variables as ScFvs; Fig. 1). In either format, the linker used for ScFv generation was not the G_4_S linker but rather the modified Bird and colleagues linker (described in Methods; ref. 23) that does not bind to GLPB30 (Fig. 2). For the TAA-binding arms, we utilized variable domains binding to either the PSMA or another prostate cancer TAA, transmembrane protein with EGF like and two Follistatin like domains 2 (TMEFF2).

## BsAb and Tumor Cells are Required for Expansion, Proliferation, and CD107a Expression In 3–23/B3 CAR T Cells

**FIGURE 4:**
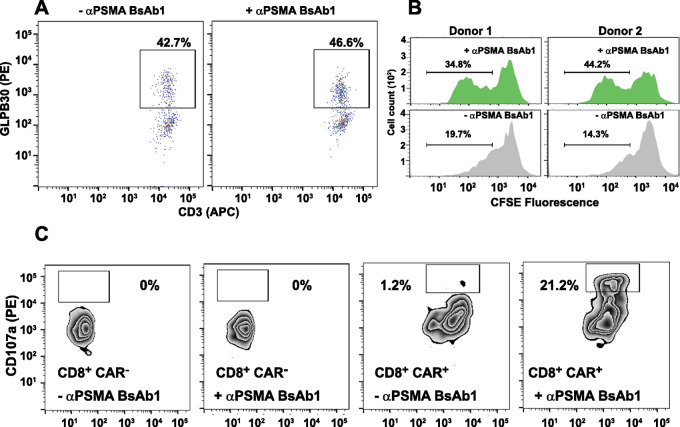
Bispecific cell binding, proliferation, and ligand engagement–dependent proliferation and degranulation. **A,** The presence of BsAb alone does not alter CAR surface expression. BsAb molecules (5 μg/mL) were added into 3–23/B3 CAR T cells with a (G_4_S)_4_ linker and incubated for 48 hours. CAR-surface expression in CD3/CD4/CD8^+^ T cells was detected by anti-G_4_S antibody (CAR in CD8^+^ T cells shown here) and remained similar in the presence or absence of BsAbs. **B,** CAR T cells generated using pan T cells from two different donors were labeled with CFSE, coculture with PSMA expressing tumor cells in presence/absence of BsAb. CFSE staining intensity was analyzed 72 hours after stimulation. CFSE staining in absence of BsAb is shown in gray histograms and those in presence of BsAb in green histogram. **C,** (G_4_S)_4_-containing CAR T cells were cocultured with PSMA-expressed tumor cells with and without BsAb1. After 5-hour coculture, CD107a detection of CAR T cells was measured. GLPB30 mAb was used to detect CAR expression. Representative plots shown CAR^−^ cells had no CD107a expression in the presence or absence of BsAb1. In the CAR^+^ population, only cells incubated with BsAb showed appreciably increased CD107a expression, suggesting BsAbs are necessary for CD107a expression in the presence of tumor cells.

We next examined whether the presence of BsAb targeting G_4_S linker affected CAR surface expression in 3–23/B3 ScFv–expressing CAR T cells in the absence of tumor cells. TAA-independent adapter CAR activation could lead to unfavorable conditions such as ligand-independent exponential expansion, constitutive cytokine release, and phenotypical changes not conducive to tumor killing (28). Cultured CAR T cells were divided and BsAb (5 μg/mL) was added into cultured CAR T cells while control wells were untreated. Cells were then extensively washed, and CAR surface expression was observed at 48 hours. As shown in Fig. 4A, incubation with soluble BsAb did not alter the surface expression level of CAR, indicating that binding of the BsAbs do not alter surface expression of the CARs.

As incubation with the BsAb alone did not alter CAR surface expression, we next examined whether the addition of tumor cells (in the presence and absence of bispecific molecule) could induce proliferation. We compared proliferation of CFSE-labeled CAR T cells in the presence or absence of BsAb with PSMA-expressing tumor cells. Our results demonstrate increased proliferation of 3–23/B3–bearing CAR T cells (observed in two donors) when BsAbs were added into coculture (Fig. 4B). Notably, some proliferation was observed in the absence of BsAb, but this is likely due to allogeneic reactions with the tumor cells.

CD107a is an effective biomarker of T-cell activation, degranulation, and cytolytic function (25). Using 3–23/B3–expressing CAR T cells, we sought to determine whether the presence of the BsAb targeting G_4_S linker and PSMA could activate CAR T cells and induce CD107a expression in the presence of PSMA^+^ tumor cells. 3–23/B3 CAR T cells were cocultured with PSMA-expressing tumor cells in the presence or absence of BsAb (5 μg/mL). After 5-hour coculture, increased CD107a expression was observed in the total cell population only in the presence of BsAb, suggesting that degranulation occurred in response to BsAb addition (Fig. 4C). GLPB30 antibody was used to detect G4S-bearing CAR T cells (after washing). For both populations of CD8^+^ cells (CAR^+^CD8^+^ and CAR^−^CD8^+^), we compared CD107a expression. As shown in Fig. 4C, in the presence of BsAb, CAR^+^CD8^+^ cells are enriched for CD107a expression (as high as 21.2% of total), while far lower levels of CD107a were observed in absence of BsAb in both the CAR^+^ populations. Moreover, CD107a expression was undetectable in CD8^+^CAR^−^ cellular population, in the presence and absence of BsAb. Our results demonstrated the increase of CD107a expression was mainly contributed by CD8^+^CAR^+^ cells in the presence of BsAbs.

### Soluble BsAbs Do Not Induce Cytokine Production in Anti-CD19 CAR T Cells

To further demonstrate that BsAbs targeting the G_4_S linker did not activate CAR T cells in the absence of cells or immobilized antigen, we utilized anti-CD19 CAR T cells that can be activated by beads immobilized with CD19 antigen. Anti-CD19 CAR T cells were incubated with either soluble recombinant human CD19 extracellular domain-Fc fusion (rhCD19), BsAb, or protein A beads loaded with rhCD19 or BsAb. After 72-hour incubation, IFNγ, IL6, and GM-CSF levels produced by CAR T cells were quantified. We observed that only bead immobilized bispecific molecules or rhCD19 antigen resulted in significantly increased cytokine production in anti-CD19 CAR T cells, whereas soluble BsAb or rhCD19 did not increase cytokine levels above the untreated control (Supplementary Fig. S2). This result is consistent with 3–23/B3 CAR T-cell data showing soluble BsAbs do not induce proliferation or activation.

## Cytotoxic Activity of 3–23/B3 CAR T Cells Is Mediated by BsAbs

**FIGURE 5:**
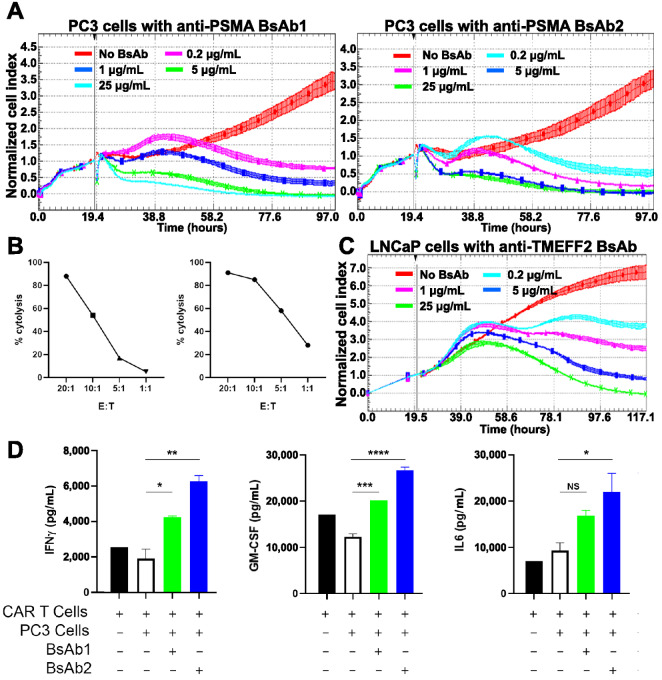
Dynamic monitoring of 3–23/B3 CAR T-cell–mediated cytotoxicity and cytokine profiling. xCelligence cytotoxicity assay was used to measure real-time tumor cell lysis by 3–23/B3 bearing CAR T cells in presence of diluted BsAbs. Bispecific anti-PSMA and anti-G_4_S linker (GLPB30) molecules (BsAb1 and 2) were generated, and further tested in xCelligence cytotoxicity assay targeting PSMA-expressing PC3 cells (**A**). In this 97-hour experiment, the E:T ratio of 3–23/B3 G_4_S-containing CAR T cells to PC3 cells was 5:1. Experiments were performed in triplicate. In a separate experiment (**B**), percent cytolysis at 72 hours at different E:T ratios were also accessed and are shown for BsAb 1 and 2 (added at 5 μg/mL). **C,** Similar xCelligence cytotoxicity experiments were performed using anti-TMEFF2 and GLPB30 bispecific antibodies. Dose dependent cytotoxicity was observed only in the presence of BsAb. **D,** In another separate experiment, cytokine profiles of supernatant in coculture of CAR T and tumor cells (E:T = 5:1) were assessed. INFγ, GM-CSF, and IL6 levels were measured at 72 hours after incubation and were elevated in the presence of BsAbs. Data are shown as the mean ± SD. Significance between groups containing both CAR T and PC3 cells were calculated using one-way ANOVA with multiple comparisons (Dunnett's test). *, *P* < 0.05; **, *P* < 0.01;***, *P* < 0.001; ****, *P* < 0.0001.

We next examined the ability of 3–23/B3 ScFv bearing CAR T cells to lyse tumor cells in the presence of BsAbs. First, BsAbs targeting PSMA and the G_4_S linker were utilized as conduit (adapter) molecules. Anti-PSMA BsAb1 contains GLPB30 Fab arms with an anti-PSMA ScFv appended to the heavy-chain C-terminus. Anti-PSMA BsAb2 uses a reverse orientation, with anti-PSMA Fab arms and a C-terminal GLPB30 ScFv. When these anti-PSMA × anti-G_4_S BsAbs were titrated in the presence of 3–23/B3 CAR T cells and PSMA^+^ PC3 cells (E:T ratio = 5:1), tumor cell lysis was observed using xCELLigence monitoring (Fig. 5A). In wells lacking BsAb, tumor cell growth continued unabated. 3–23/B3 CAR T cells mediated tumor cell–specific lysis in the presence of either BsAb. Further analysis of this cytotoxicity experiment can be found in Supplementary Fig. S3 where cytotoxicity at the 96-hour timepoint is quantified and statistically significant cytotoxicity is observed at all concentrations of BsAb (compared with the untreated cells).

In a separate experiment, we varied E:T ratio of CAR-T:PC3 cells with a fixed BsAb concentration (5 μg/mL). These results show that 3–23/B3 CAR T BsAb–specific killing varies with E:T ratio (Fig. 5B). Similar results to Fig. 5A were observed using an anti-TMEFF2 BsAb containing anti-TMEFF2 Fab arms with the GLPB30 ScFv appended to the heavy-chain C-terminus. In this case, 3–23/B3 CAR T cells were shown to kill LNCaP (a prostate-derived tumor cell line) in the presence of BsAb (Fig. 5C). Cytotoxicity and statistical significance of these TMEFF2-positive LNCaP cells is also shown (at the 96-hour timepoint) in Supplementary Fig. S3.

Having demonstrated the cytotoxic potential of conduit CAR T cells in the presence of BsAbs, we next sought to quantify whether cytokines commonly observed upon CAR T-cell activation were also being produced in the presence of BsAbs. IFNγ, IL6, and GM-CSF levels produced by CAR T cells were quantified by Intellicyt iQue measurement. CAR T cells were cocultured with tumor cells at E:T ratio of 5:1 and bispecific molecules were added at a final concentration of 5 μg/mL. The addition of bispecific molecules significantly increased cytokine production by CAR T cells in the presence of target cells (Fig. 5D). We observed IFNγ levels more approximately double in the presence of BsAb1 and triple in the presence of BsAb2. GM-CSF levels increased similarly (compared with CAR T and PC3 alone). IL6 levels were significantly increased in the presence of BsAb2, but not BsAb1.

### Cytotoxic Activity of Anti-CD19 CAR T Cells Redirected to Target PC3 Cells

**FIGURE 6:**
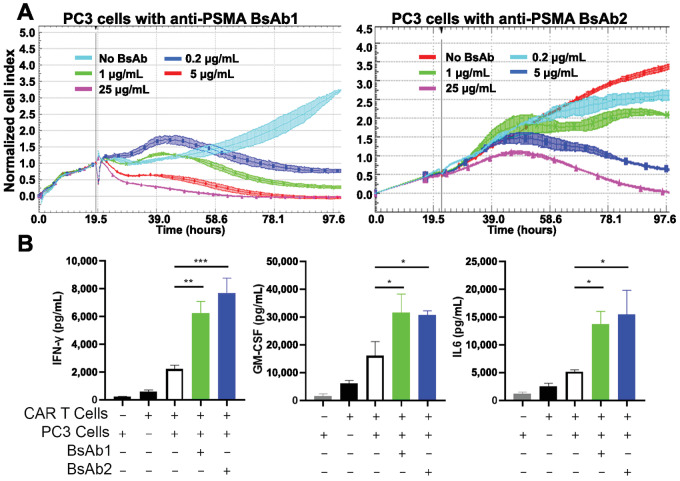
Dynamic monitoring of anti-CD19 CAR T-cell–mediated cytotoxicity and cytokine profiling. Real-time tumor cell lysis by anti-CD19 CAR T cells redirected by BsAbs to target PSMA^+^ cells was measured using xCelligence monitoring. **A,** Bispecific anti-PSMA and anti-G_4_S linker (GLPB30) targeting molecules (BsAb1 and 2) were added to wells at the 19-hour timepoint. Cytotoxicity curves for various BsAb concentrations are shown. **B,** In a separate experiment, cytokine profile of supernatant in coculture of CAR T and tumor cells (E:T = 5:1). INFγ, GM-CSF, and IL6 levels were assessed at 72 hours after incubation and were elevated in the presence of BsAbs. Data are shown as the mean ± SD. Significance was calculated using one-way ANOVA with multiple comparisons (comparing to CAR T + PC3, no BsAbs; Dunnett test). *, *P* < 0.05; **, *P* < 0.01; ***, *P* < 0.001; ****, *P* < 0.0001.

After demonstrating G_4_S-targeting bispecifics could redirect 3–23/B3–bearing CAR T cells, we next examined whether they could be used to redirect CD19-targeting CAR T cells to target a novel antigen. We again utilized BsAb1 targeting PSMA and the G_4_S linker as conduit or adapter molecules. BsAb1 and 2 were again titrated in the presence of MYC-tagged anti-CD19 CAR T cells [bearing a (G_4_S)_3_ ScFv linker] and PSMA^+^ PC3 cells (E:T ratio = 5:1), tumor cell lysis was again observed using xCELLigence monitoring (Fig. 6A). In wells lacking BsAb1 or 2, tumor cell growth continued unabated, whereas cells treated with BsAb1 were able to lyse the PSMA^+^ PC3 cells. Interestingly, BsAb1 seemed to show better killing using the CD19-targeting CAR T cells than BsAb2. Further analysis of this cytotoxicity experiment can be found in Supplementary Fig. S3.

Having demonstrated the cytotoxic potential of conduit CAR T cells in the presence of BsAbs, we next sought to quantify whether cytokines commonly observed upon CAR T-cell activation were also being produced in the presence of BsAbs. IFNγ, IL6, and GM-CSF levels produced by CAR T cells were quantified. CAR T cells were cocultured with tumor cells at E:T ratio of 5:1 and bispecific molecules were added at a final concentration of 5 μg/mL. The addition of bispecific molecules significantly increased cytokine production by CAR T cells in the presence of target cells (Fig. 6B). We observed significant increases in IFNγ, GM-CSF levels, and IL6 levels in the presence of BsAb1 and 2.

## Discussion

CAR T-cell therapy has proven remarkably effective in treating certain hematopoietic cancers (7). To further refine and advance this transformative therapy, researchers now face the multipronged challenges of understanding and reducing relapse rates, expanding this cell therapy to solid tumors, managing serious adverse events, and lowering costs. One approach that has been proposed to address some of these challenges is to use soluble “adapter” molecules to act as a conduit between CAR T cells and target tumor cells. Most current clinical CAR T-cell candidates, and all the FDA-approved CAR T-cell therapies target one TAA via a fixed, membrane-bound antibody fragment. A drawback of this fixed design is tumors that can downregulate expression or alter the structure or localization of the targeted antigen (antigen escape) can experience relapse when their exogenous CAR T-cell population can no longer engage tumor cells (8). An adapter CAR T-cell approach could offer more flexibility in which TAAs could be targeted and allow for dosing control to reduce toxicity. Our conduit CAR T-cell adapter described herein targets the (G_4_S)_n_ linker present on many existing clinical CAR molecules. The (G_4_S)_n_ linker consists of a repeated G_4_S sequence and is commonly used to connect VH and VL domains in ScFvs. CAR constructs and other biologic therapeutics utilizing this small (∼25 kDa) antibody domain usually contain at least one exposed G_4_S linker [typically (G_4_S)_3_ or (G_4_S)_4_]. Indeed, clinically approved CARs Kymriah (tisagenlecleucel); and Yescarta (axicabtagene ciloleucel) utilizing the FMC63 ScFv contain (G_4_S)_3_ linkers.

In this study, we generated and characterized a mAb (GLPB30) that can specifically target the G_4_S linkers and generated bispecific molecules targeting both TAAs and this ScFv linker. These bispecific antibodies were capable of inducing CAR T-cell proliferation, and activating CAR T cells in the presence of BsAb-coated beads or BsAb-treated tumor cells (but not soluble BsAb alone). In addition, we were able to demonstrate redirected killing of PSMA^+^ cells using both CD19-targeting CAR T cells as well as 3–23/B3–bearing CAR T cells (lacking inherent TAA specificity).

Use of this G_4_S-targeting adapter approach could allow for existing CAR T-cell therapies to be redirected to different TAAs upon relapse. Patients experiencing relapse due to loss or downregulation of CAR-targeted antigen (e.g.*,* CD19), could be treated with G_4_S binding adapters to target additional TAAs. Alternatively, bispecific adapters could be utilized during initial CAR treatment, prior to any potential relapse. Utilizing adapter bispecifics while CARs are newly primed and activated may expand the target cell population and reduce the odds of relapse due to antigen loss or downregulation. Currently, all CD19-targeting CARs approved by the FDA have G_4_S linkers that can be targeted via an anti-G_4_S linker adapter molecule. If other ScFv linkers are used for future CARs, applying this approach to those CARs would of course require the generation of new reagents specific for those linkers. A methodology has been described using CD19 antigen fused to TAA-targeting moieties to redirect CD19-targeting CARs (29). This approach is of course limited to use with CD19 CARs. A G_4_S linker targeting adapter molecule may be more broadly applicable to any G_4_S-containing CAR T-cell therapeutics.

In addition to use in conjunction with existing CAR T-cell therapies, G_4_S-targeting adapter molecules could be used with CARs lacking affinity to any TAA (as described herein with the 3–23/B3 bearing CAR). An advantage of this approach is reduced safety concerns as the presence of an adapter or conduit bispecific molecule controls CAR T-cell proliferation and activation. By halting dosing of the adapter molecule, CAR proliferation and activation will stop (once the adapter is cleared), thus eliminating the need for a suicide gene switch engineered into some novel CAR T-cell therapeutics (30).

Several adapter CAR designs have been proposed, each with various potential advantages and disadvantages (14, 31–34). The reliance on nonhuman proteins for an adapter or CAR molecule such as the yeast transcription factor GCN4 (22) or *Streptococcus pyogenes* derived SpyCatcher/SpyTag systems (31) could be disadvantageous due to potential immunogenicity. Although the G_4_S linker itself (a sequence not found in the human proteome) could potentially pose an immunogenicity risk as well, this has yet to be observed in multiple approved clinical therapeutics bearing this linker. Nevertheless, there is of course the chance of immunogenicity or an anti-drug antibody (ADA) response to the linker that may interfere with this approach. Indeed, preexisting ADA to G_4_S linker–containing ScFvs has been observed before (although it is unclear whether the G_4_S linker was the epitope; ref. 35) and O-linked glycans on G4S linkers (a heterogeneity concern) could also pose an immunogenicity risk (36).

Prior to clinical studies, further refinement of the conduit CAR approach will be necessary. In this study, we used a rabbit-derived anti-G_4_S variable domain, but for clinical purposes, this molecule would likely be humanized to further reduce immunogenicity. Another step before clinical validation, would be to confirm a lack of off-target binding of the G_4_S binding antibody. This will be needed to ensure the adapter BsAb will have specificity for only linkers displayed on the CAR surface. Furthermore, a conduit CAR adapter approach will likely be limited clinically to patients not being cotreated with additional G_4_S-containing therapeutics as these may interfere with conduit–CAR T-cell targeting. While this study provides initial proof of concept for an ScFv-linker targeting adapter CAR *in vitro*, multiple *in vivo* studies using appropriate xenograft or syngeneic models may be required to refine and validate this concept further. COVID-19–related resource constraints and new prioritizations prevented the completion of lengthy *in vivo* studies for this project. Nevertheless, due to the rapid evolution and impact of work within the CAR T field, we strongly believe it is very timely to present this emerging strategy for improving the specificity and utility of CAR T cells for treating cancer. We believe comprehensive *in vivo* models are required to advance this concept and prior to clinical development of programs using this technology, yet that was unfortunately not possible due to current constraints mentioned above.

Conduit CAR (along with other adapter or universal CAR formats), offer the advantages of being able to fine tune and control CAR function to quickly meet emerging clinical understanding of optimal CAR properties. It has recently been shown that decreasing affinity of CAR molecules can increase proliferation and antitumor activity in a clinical setting using anti-CD19 CAR T cells (37). Adapter CARs, such as the system described in this study, allow for tuning of affinity and avidity of adapters to explore optimal properties for the cytotoxic immune synapse via changing intrinsic affinity, stoichiometry, and valency.

## Supplementary Material

Supplementary Figures 1-3 Supplementary Figure 1. Bispecific GLPB30 x anti-PSMA constructs run on an SDS page gel under non-reduced and reduced conditions. Supplementary Figure 2. Cytokine profiles of anti-CD19 CAR-T supernatants. Supplementary Figure 3. 96-hour CAR-T and BsAb cytotoxicity analysis.
