# Supplementary material for: Conduit CAR: Redirecting CAR T-Cell Specificity with A Universal and Adaptable Bispecific Antibody Platform
Source: Cancer Res Commun. 2022 Mar 22;2(3):146–57. doi: 10.1158/2767-9764.CRC-21-0150 (PMC9980914; doi:10.1158/2767-9764.CRC-21-0150)
Supplement: Supplementary Figures 1-3 — Supplementary Figure 1. Bispecific GLPB30 x anti-PSMA constructs run on an SDS page gel under non-reduced and reduced conditions. Supplementary Figure 2. Cytokine profiles of anti-CD19 CAR-T supernatants. Supplementary Figure 3. 96-hour CAR-T and BsAb cytotoxicity analysis. [file crc-21-0150-s01.docx]

**Conduit CAR: A Universal CAR-T Platform for Adaptable Specificity**

**Supplementary Figures**


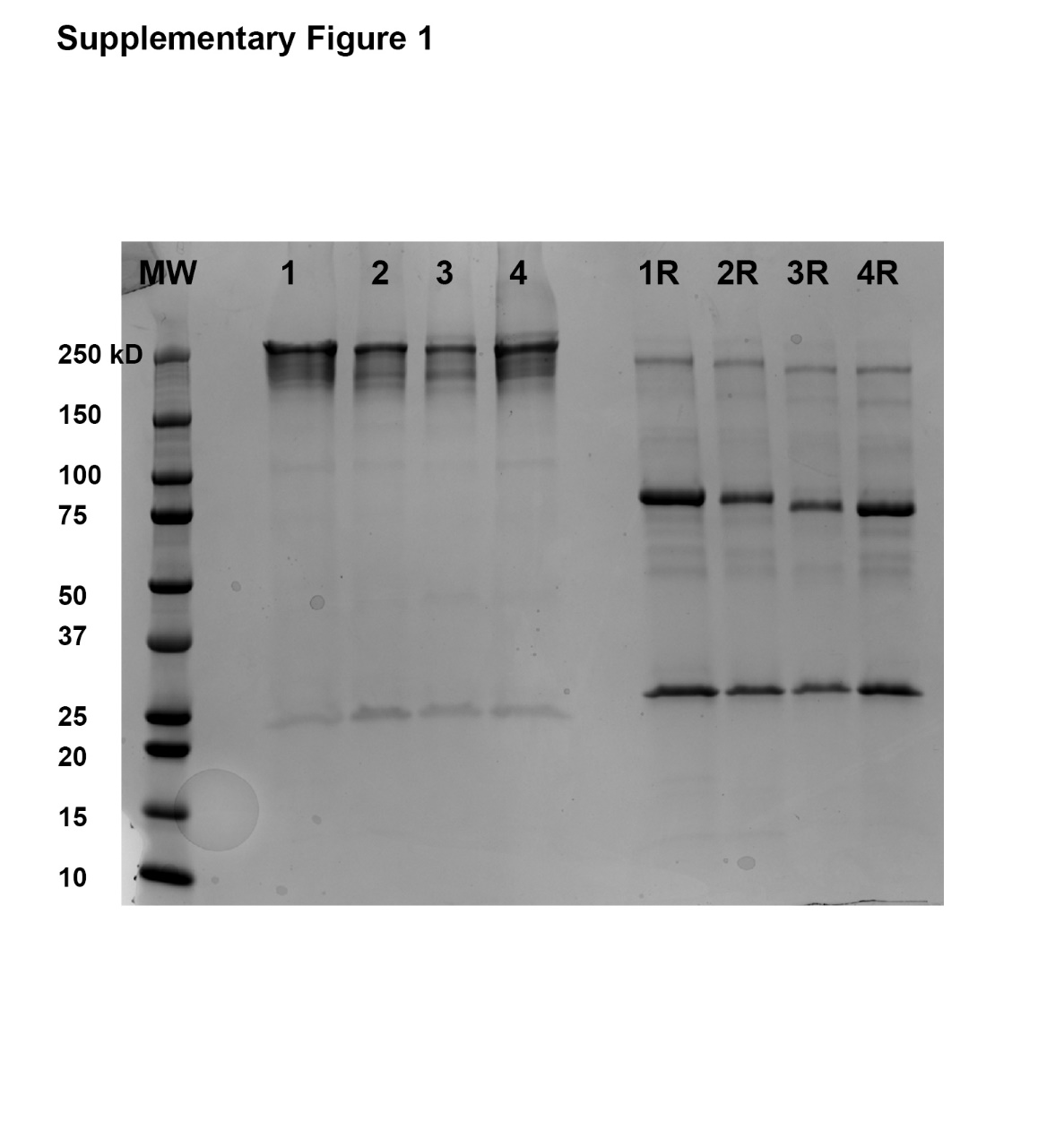


**Supplementary Figure 1**. Bispecific GLPB30 x anti-PSMA constructs were run on an SDS page gel under non-reduced and reduced conditions. All 4 bispecifics are formatted with an ScFv linked to the C terminus (first described by Coloma et al.^24^). Constructs 1 & 2 have anti-PSMA as the C-terminal ScFv (1 denotes the Scfv in the “Heavy-Light” (H-L) orientation and 2 denotes the “Light-Heavy” L-H orientation) with GLPB30 Fab arms. Constructs 3 & 4 have GLPB30 as the C-terminal ScFv (3 is in H-L orientation and 4 is in L-H orientation) with anti-PSMA Fab arms. Antibodies 1 and 3 were used in cytotoxicity assays (Bs1 & Bs2 respectively).

**Supplementary Figure 2**


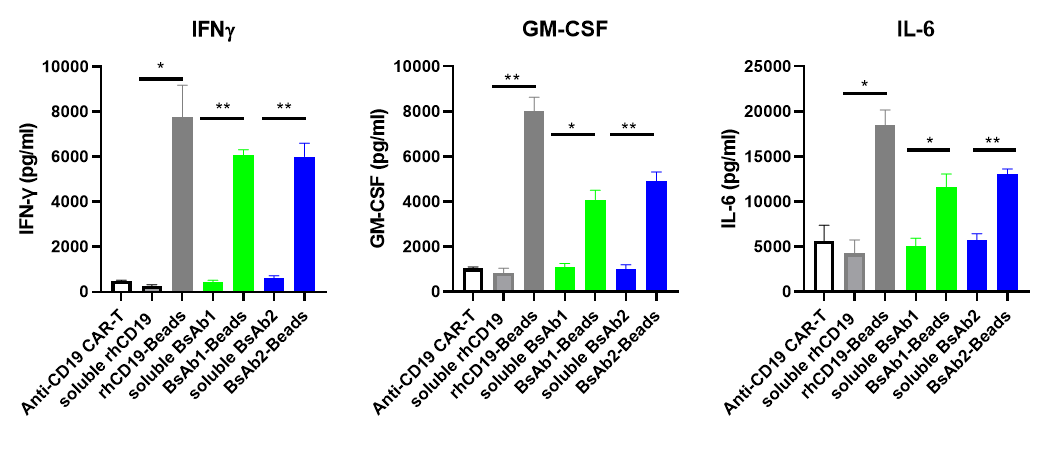


**Supplementary Figure 2**. Cytokine profiles of anti-CD19 CAR-T supernatants. INFγ, GM-CSF and IL-6 levels were assessed at 72 hours after incubation with recombinant 5 μg/ml human CD19-Fc (rhCD19), Bispecific antibodies, or protein A beads coated with those reagents. In the absence of clustering via beads, neither soluble CD19 nor bispecific antibodies promoted cytokine production (i.e. not significance compared to anti-CD19 CAR alone in all panels; assessed using one-way ANOVA using multiple comparison analysis). Data are shown as the mean ± SD. Significance between soluble antigen or antibodies and their bead bound counterparts were calculated using an unpaired T-test, *p<0.05, ** p<0.01,*** p<0.001, ****p<0.0001.

**Supplementary Figure 3**


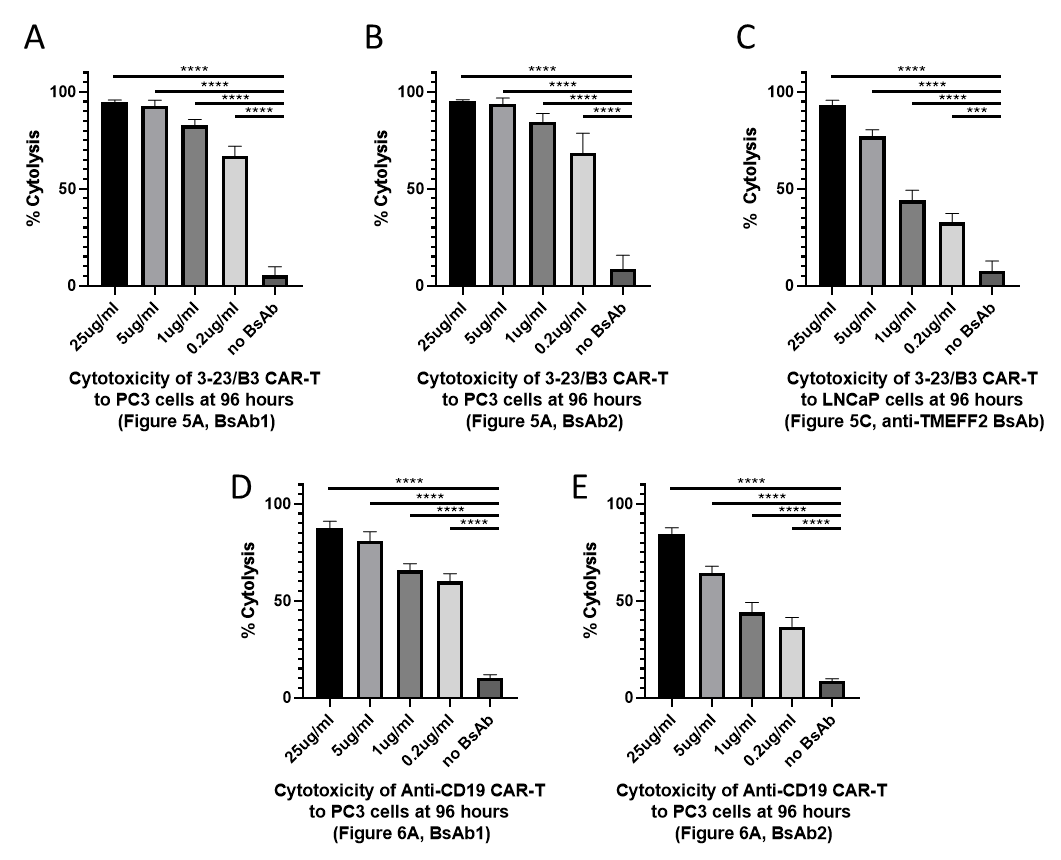


**Supplementary Figure 3**. 96-hour CAR-T and BsAb cytotoxicity analysis. At the 96-hour timepoint, of xCELLigence monitoring, percent cytotoxicity was assessed by normalizing experimental wells to “no CAR-T/No BsAb” treated wells (0% cytotoxicity) (see methods). This allowed for quantification and determination of significance of the cytotoxicity with various BsAb concentrations. This analysis was performed for (A) 3-23/B3 CAR-T mediated cytotoxicity in the presence of anti-PSMA BsAb1, (B) 3-23/B3 CAR-T mediated cytotoxicity in the presence of anti-PSMA BsAb2, (C) 3-23/B3 CAR-T mediated cytotoxicity in the presence of anti-TMEFF2 BsAb1, (D) Anti-CD19 CAR-T mediated cytotoxicity in the presence of anti-PSMA BsAb1, and (E) Anti-CD19 CAR-T mediated cytotoxicity in the presence of anti-PSMA BsAb2. Data are shown as the mean ± SD. Significance between “No BsAb” controls and BsAb treated cells was calculated using one-way ANOVA with multiple comparisons (Dunnet’s test), *p<0.05, ** p<0.01,*** p<0.001, ****p<0.0001.
